# Items, Selections and Remarks

**Published:** 1870-04

**Authors:** W. W. Miner


### Items, Selections and Remarks.

BY W. W. MINER, A. B.

Transfusion was recently performed in Dublin, by Drs. Beatty, Colles and McDonald, on account of uterine hemorrhage, and with complete success. Two more quite successful cases of transfusion are reported from Heidelberg.——The common carotid artery, in a person who had been stabbed, was recently ligated by Dr. Marquardt, of Thorne, Germany. The ligature came away in twenty days and the patient fully recovered.——Prof. W. H. Byford, of Chicago, recently removed a horse-shoe pessary two and one-half inches long and one and three-fourths inches from side to side, from the bladder of a young married lady, where it had been inserted through the mistake of a physician and where it had remained three months.——Dr. Mason exhibited to the New York Pathological Society, Feb. 9th, the skeleton foot of a dissecting-room subject, with two internal cuneiform bones. He had only been able to find three similar cases on record.—*Record.*——Dr. J. Buchser reported a short time since in the *Record*, a successful case of transfusion. Three ounces of defibrinated blood were injected into the median basilic vein of a lady who was in the convalescent stage of typhoid fever, but whose respiration numbered sixty per minute and her pulse one hundred and forty. The operation lasted one-half an hour and was followed by immediate improvement.

An effort is being made to establish a Medical Society in East Virginia.——A move is being made in San Francisco, Sacramento and Oakland, towards inviting the National Medical Convention to meet in San Francisco next year.——The annual meeting of the Illinois State Medical Society, will take place at Dixon, Lee County, Tuesday, April 17th.——The second annual meeting of the Minnesota State Medical Society was held at St. Paul, Feb. 2.

The "New York State Hospital for Diseases of the Nervous System," recently incorporated, has for its object the gratuitous treatment of "indigent persons who may be affected with acute or chronic diseases of the nervous system, especially epilepsy and paralysis, but excluding insanity;" and clinical instruction to physicians and students in such branches of medicine. Dr. Wm. Hammond holds the position of physician-in-chief; Dr. Vance as assistant physician, while Drs. Flint, Ellicott, Wood and Sayre, constitute the consulting board.——The directors of the Mount Sinai Hospital, New York City, have decided to erect a new building on Lexington avenue whose cost is to be $300,000, and the corner stone of which is to be laid in May.——An association of the Alumni of the Jefferson Medical College has been formed, of which, Prof. S. D. Gross is President, and Dr. R. J. Dunglison, Corresponding Secretary.

The physician who attended Count Bismarck's son, lately wounded in a duel at Bonn, paid 160 visits to the young man. When he had ceased his visits the countess tendered him $25.00!! He demanded 160 thalers ($120.00.) His very moderate bill was refused. Next time he had better demand cash each visit.——The *Scientific American* states respecting a mis-statement which was somewhat current, concerning Prof. Agassiz:—"The Professor denies saying 'he did not want any who believed in the first chapter of Genesis to attend his lectures at Harvard College.' In a letter to a friend he writes, 'I seldom notice such things; it is often useless to do so, and habit has made me somewhat callous to misrepresentations, but something in the tone of your letter makes me unwilling to leave it go unanswered. I said, theological interpretation of Genesis giving 6,000 years as the age of the world, was contrary to geological evidence, and any one subservient to that idea in his remarks, could not be a geologist.'" The *Nashville Medical Journal* adds that to contend for the literal meaning of Genesis, would be as absurd as to give no credence to it.

The Chicago Medical College conferred last month the degree in medicine upon twenty graduates, the ad eundum degree upon four, among whose names is that of Mary H. Thompson, M. D., and honorary degrees upon three gentlemen.——The Rush Medical College, in February last, conferred the degree of M. D. upon one hundred and thirty graduates; the ad eundum degree upon four and the honorary degree upon one.——Alumni Associations of both the Chicago and Rush Medical Colleges seem to be quite heartily engaged in and appear to be of value in establishment of intercourse and good will among physicians——The twenty-fourth annual meeting of the Superintendents of Hospitals for Insane, is to be held at the Allyn House, Hartford, Conn., June 10th.——Col. Emmons Clark was chosen Secretary of the New York Board of Health, April 20th, and Dr. Moreau Morris, City Sanitary Superintendent, in place of Dr. Harris, the previous incumbent.

It will be seen from the *Dental Cosmos* that the degree of Doctor of Dental Surgery (D. D. S.,) has been conferred, within the last two months, by the Philadelphia Dental College upon forty-one graduates; by the Pennsylvania College of Dental Surgery upon thirty-eight graduates and three practitioners; by the Baltimore College of Dental Surgery upon twenty-four graduates; by the New Orleans Dental College upon five graduates and several honorary recipients. The Dental School of Harvard University conferred, March 9th, the degree of Doctor of Dental Medicine (D. M. D.,) upon twelve graduates.

F. B—— writes us if the hypodermic injection of Ammonia proves useful in cases of snake bite as seem to be affirmed by Prof. Halford's experiments, its use in hydrophobia would also be likely to be of service. We notice that B. Wills Richardson, in a case of poisoning from one drachm and one half of tincture aconite, made hypodermic injection of one-half drachm of liquor ammonia at three times, and the patient slowly recovered; he suggests the trial of this agent in hydrophobia and also in chloroform poisoning.——Sir Henry Thompson, in a recent lecture, gave his opinion in regard to the endoscope. He considers it almost valueless. In nineteen cases out of twenty, it is unnecessary and in the twentieth, difficult of management, and a source of pain to the patient. He does not utterly repudiate it, but his testimony will give it a quietus. He says that it is essentially the same instrument as that used by Mr. Avery, of London, twenty-five years ago.—*Richmond Medical Journal.*

Dr. Andrews, a London chemist, announces that he has experimentally demonstrated that the transition of carbonic acid from the state of a gas to that of a liquid is a continuous process.——Italian naturalists propose to effect a reform in the chemical nomenclature of organic bodies, which subject comes before their next congressional meeting this year..——The *Reporter* says that a new and very delicate test for arsenic has been discovered by Bettendorf. Its sensibility is so great that it is said to be capable of detecting one part of arsenic in a million parts of solution; and the presence of antimony does not affect it. In order to apply this test, the arsenious, or arsenic liquid is mixed with aqueous hydric chloride (hydrochloric acid), until fumes are apparent; thereupon stannous chloride is added, which produces a basic precipitate, containing the greater part of the arsenic as metal mixed with stannic oxide.

Dr. Ambrose Tardieu is well known all over the world as an expert and a teacher of forensic medicine. But he is not so devoted to the science that it prevents him from being a violent Bonapartist. When Prince Pierre Bonaparte murdered Victor Noir, the first physician summoned was a general practitioner in the immediate vicinity. He was replaced in a few hours by Dr. Tardieu in his official character as an expert. Dr. Tardieu declined all assistance or information from the general practitioner, and made up his report solely from his own observation. More than this, his expressions and report were plainly intended—so at least, all who differ from him in politics say— to shield the prince from the results of his crime. On his appearance in the lecture room after the trial, the students hooted and hissed him to such a degree that he said he would resign his chair. Since then the school has been closed till the first of May by the government.—*Phila. Reporter.*

M. Poiseuille, the inventor of the hæmodynamometer, has lately died in Paris at the age of seventy-one.——Prof. Helmholtz has been elected corresponding member of the Paris Academy of Science.——Dr. D. Hayes Agnew has been appointed to the newly established chair of Clinical and Operative Surgery in the Medical Department of the University of Pennsylvania, and Dr. H. L. Hodge, Jr., has been appointed Demonstrator of Anatomy, in the same institution, a position which Dr. Agnew has lately resigned.——Dr. J. C. Peters has terminated his editorial connexion with the New York Medical Gazette..——Professors Hun, Quackenbush, Vanderpoel and Mosher have resigned their positions in the Albany Medical College.

Prof. Billroth, of Vienna, has witnessed 12,500 cases of anæsthesia from chloroform and has seen but one death from it.——M. Jonon, a professor at Nantes, has successfully performed ovariotomy on a patient aged twelve years.——Small Pox is epidemic, at the rate of 90 to 100 deaths per week, in Paris. —*Gazette*.——Dr. John H. Packard, recently presented before the College of Physicians of Philadelphia, a specimen of united intra-capsular fracture of the neck of the femur. The specimen was referred to a committee, who reported:—" After careful study of the specimen, we are convinced that it is one of bony union of an impacted intra-capsular fracture of the cervix femoris." ——A New York coroner's jury found a verdict, April 5th, of death, in a case of delirium tremens, from congestion of the brain occasioned by the overdosing of morphine by a physician who also wrote "essentia somnolentia" in his prescription, meaning morphine; and this private nomenclature he was in the habit of using so as to obtain his percentage from the druggist who was "doing his prescription business."

